# HPTLC Analysis of Bioactivity Guided Anticancer Enriched Fraction of Hydroalcoholic Extract of *Picrorhiza kurroa*

**DOI:** 10.1155/2015/513875

**Published:** 2015-10-18

**Authors:** Md. Nasar Mallick, Mhaveer Singh, Rabea Parveen, Washim Khan, Sayeed Ahmad, Mohammad Zeeshan Najm, Syed Akhtar Husain

**Affiliations:** ^1^Bioactive Natural Product Laboratory, Department of Pharmacognosy and Phytochemistry, Faculty of Pharmacy, Jamia Hamdard, New Delhi 110062, India; ^2^Human Genetics Laboratory, Department of Bioscience, Jamia Millia Islamia, New Delhi 110025, India

## Abstract

*Objective*. Hydroalcoholic extract of *Picrorhiza kurroa* and its fractions were subjected to *in vitro* screening for cytotoxicity; further best active fraction (BAF) obtained was tested against Ehrlich ascites carcinoma (EAC) model in Balb/c mice after its quality control analysis. *Methods*. Cytotoxicities of all the fractions and mother extract of *P. kurroa* were determined, using MTT assay on breast cancer (MCF-7, MDA-MB 231) and cervical cancer (HeLa, SiHa) cell lines. Metabolic fingerprinting was developed using HPTLC with quantification of biomarkers (cucurbitacins B and E; betulinic acid; picrosides 1 and 2; and apocynin) in BAF. The EAC tumor-bearing mice were used for *in vivo* anticancer activity after oral administration (50 mg Kg^−1^) for 10 days. *Results*. Cytotoxicity assay of mother extract and its fractions over breast cancer and cervix cancer cell lines showed that dichloromethane (DCM) fraction was most cytotoxic (IC_50_ 36.0–51.0 *µ*g mL^−1^ at 72 h). Oral administration of DCM fraction showed significant reduction in tumor regression parameters, viable tumor cell count and restoration of hematological parameters may be due to presence of cucurbitacins B and E; betulinic acid; picrosides 1 and 2; and apocynin, as compared to the untreated mice of the control group. *Conclusion*. The DCM fraction of *P. kurroa *displayed potent anticancer activity and can be further explored for the development of a potential candidate for cancer therapy.

## 1. Introduction

Cancer is a major public health problem in many parts of the world. It is currently the second leading cause of death and is expected to surpass heart diseases as the leading cause of death in the next few years [1]. Normal tissue homeostasis is maintained by the two counterparts, namely, cell proliferation and apoptosis [2]. Failure of apoptosis mechanism may result in limitless growth and division of cells. The conventional therapies for cancer include chemo- and radiotherapies mediated by inducing apoptosis or inhibiting proliferation in neoplastic cells [3]. These therapies cause damage to healthy tissues around the tumors [4, 5] and also develop resistance by numerous tumors [6]. Researchers have been studying alternatives of cancer therapy by applying potential biological molecules to target neoplastic tumors [7].

Plant-based immunomodulators are nowadays receiving adequate attention and have been evaluated for their active potential to modulate immune responses [8, 9]. Many of the natural products are in clinical use [10]. Identification of the active components and molecular basis for the action of a traditional medicine is likely to make natural products more acceptable for humans, an approach sometimes referred to as Reverse Pharmacology [11].

*Picrorhiza kurroa* Royle ex Benth. is a well-known herb of the traditional systems of medicine. It is a perennial herb belonging to the family Scrophulariaceae and found in the Himalayan region at an altitude of 3000–5000 m [12]. The rhizome of* P. kurroa* is traditionally used for liver disorders and is known to be DNA protective [13] and antioxidant [14]. The rhizome has been reported to contain iridoid glycoside like picroside 1, picroside 2 [15–17]; terpene like cucurbitacins [18–20]; and flavonoids like apocynin [21], which are responsible for the anticancer potential of the plant [18, 20, 22–33]. The present investigation was designed to investigate the cytotoxic potential of hydroalcoholic (mother) extract and its bioactivity guided polar and nonpolar compound enriched fractions, in this case, hexane (fat-rich fraction), dichloromethane (DCM) (terpenoid- and flavonoid-rich fraction), butanol (glycoside-rich fraction), and acetone (tannins- and phenol-rich fraction), whereas methanol and water contain the remaining polar compounds.

The most cytotoxic fraction, that is, best active fraction (BAF), was further evaluated for* in vivo *anticancer potential after its quality control analysis, using HPTLC. The contents of picrosides 1 and 2, betulinic acid, cucurbitacins, and apocynin were quantified in BAF since it was terpenoid enriched fraction (DCM). The contents of one flavonoid and 6 terpenoid markers were quantified using newly developed and validated simultaneous HPTLC methods for the first time in any medicinal plant. The anticancer potential of these compounds have already been reported separately [18, 20, 22–33]; however no work has been reported till date on terpenoid enriched fraction in totality for* P. kurroa*.

## 2. Materials and Methods

### 2.1. Chemicals

RPMI-1640, phosphate buffered saline (PBS), fetal bovine serum (Gibco, USA), trypsin-EDTA, trypan blue, penicillin-streptomycin and amphotericin, and dimethyl sulfoxide (DMSO) were of molecular biology grade. Apocynin, cucurbitacin B and 3-(4,5-dimethylthiazol-2-yl)-2,5-diphenyltetrazolium bromide (MTT), and betulinic acid were obtained from Sigma-Aldrich, USA, whereas cucurbitacin D, cucurbitacin E, and picroside 1 were obtained from Chromodex, USA. Picroside 2 was provided by Sami Labs Ltd., Bangalore, India, as a gift sample. All the reference standard markers used had more than 98% purity.

### 2.2. Plant Material

Plant samples were procured from local drug market of Delhi, India, and the specimen was authenticated by a botanist, Dr. H. B. Singh, Scientist F and Head, Raw Material Herbarium and Museum, NISCAIR, New Delhi (Ref. NISCAIR/RHMD/Consult/-2010-11/1563/161/27/10-10).

### 2.3. Hydroalcoholic Extract (Mother Extract) and Its Fractionation

The 500 gm of powdered* P. kurroa* rhizome was extracted with 70% alcohol in Reflux extractor for five hours on water bath and filtered. The filtrate was evaporated to dryness under reduced pressure. The hydroalcoholic (mother) extract thus obtained was suspended in double distilled water (1 gm/10 mL) and sonicated for 15 min at 45°C. Prepared aqueous suspension was partitioned with equal proportions of hexane, DCM, and n-butanol (thrice each). The aqueous suspension left after partitioning was evaporated to dryness and the residue was sonicated further with acetone and methanol separately for 20 min, thrice each. The remaining residue and solvent fractions obtained were evaporated to dryness under reduced pressure. The extractive values and % yields of different fractions were calculated and stored at 4°C for bioactivity and quantitative analysis.

### 2.4. Cell Line and Cell Culture

All cell lines (MCF-7, SiHa, Hela, and MDA-MB 231) used in the study were obtained from National Centre for Cell Science (NCCS) at Pune, India. The cell lines were grown as monolayer cultures in RPMI-1640 media with 10% foetal calf serum (FCS) and 1% PSA (penicillin, streptomycin, and amphotericin) in a humidified atmosphere of 5% CO_2_ at 37°C.

### 2.5. Cytotoxicity Assay of* Picrorhiza* Extract and Its Fractions

The cytotoxicity assays of mother extract and its hexane, DCM, n-butanol, acetone, methanol, and water fractions were carried out to find out the best active fraction (BAF). The stock solution was prepared by dissolving 500 mg of each extract/fraction in dimethyl sulfoxide (DMSO) and volume was made up to 10 mL in volumetric flask, separately. These solutions were passed through 0.45 *μ*m membrane filter and stored at 4°C until used. These were diluted fifty times using RPMI-1640 media (1 mL to 50 mL) to get a concentration of 1000 *μ*g mL^−1^ of every extract/fractions. Further, these solutions were passed through 0.22 *μ*m membrane filter in aseptic condition before using for* in vitro* activity on different cell lines. Similarly, DMSO control was also prepared and used for every cell line.

In brief, MTT [3-(4,5-dimethylthiazole-2-yl)-2,5-diphenyl tetrazolium bromide] assay was performed on MCF-7, SiHa, HeLa, and MDA-MB 231 cell lines [34]. 1 × 10^4^ cells were seeded on 96-well plates supplemented with 100 *μ*L of the respective culture media for a period of 24 h at 37°C. It was then substituted by 100 *μ*L of fresh media containing varying concentrations of the extract/fractions (3.9, 7.8, 15.6, 31.2, 62.5, 125, 250, and 500 *μ*g mL^−1^). The plates were again incubated for 24, 48, and 72 h, separately at 37°C, by changing fresh media containing extracts/fractions every 24 h. After incubation period media were removed and fresh media added; 20 *μ*L of MTT reagent prepared in respective media (5 mg mL^−1^) was then added to all the wells. This was followed by incubation for 3 h. After seeing purple color precipitation which was very well visible under microscope, media were carefully discarded for solubilization of formazan crystals (MTT formazan). Further, 100 *μ*L of DMSO was added to each well and cells were incubated in dark at room temperature for 1 h. The purple color developed was measured at 570 nm by a microplate reader (Bio-Rad, USA). The percentage of cytotoxicity of these extracts was calculated by using the following formula:

(1) $$\begin{array}{c} \left\{\;\frac{A_{c}-A_{s}}{A_{c}}\;\right\}\times 100, \end{array}$$

where *A*_*c*_ is the absorbance of the control and *A*_*s*_ is the absorbance of the sample.

### 2.6. HPTLC Analysis

HPTLC fingerprints of mother extracts and their fractions like hexane, DCM, n-butanol, acetone, methanol, and water were carried out for their quality control and determination of number of compounds present in them. Presence of picrosides [15–17], cucurbitacins [18–20], apocynin [21], and their anticancer potential [18, 20, 22–34] has already been reported and hence simultaneous analysis of these compounds in the BAF was carried out using newly developed HPTLC methods. These methods were developed as per ICH guidelines, similar to several methods reported by the Laboratory for Quality Control of Herbal Drugs and Botanicals [35, 36].

### 2.7. Sample Preparation and Chromatographic Conditions

The dried mother extract and fractions (100 mg each) of* P. kurroa* were reconstituted using HPLC grade methanol in a 10 mL volumetric flask to get 10 mg mL^−1^ solution. These were sonicated and filtered through 0.22 *μ*m syringe filter before being used for HPTLC analysis. The samples were applied in triplicate (8.0 *μ*L each) and the width of the track was kept to 4.0 mm on precoated silica gel 60 F_254_ plates (E. Merck, 0.20 mm thickness), using Linomat V (HPTLC sample applicator). Linear ascending development was carried out in 10 × 20 cm twin trough-glass chamber (Camag, Muttenz, Switzerland). The optimized chamber saturation time for solvent system was 30 min at 25°C and relative humidity of 60%. The chromatogram was developed up to 85% of total TLC plate height. Developed chromatograms were scanned at 254 nm for DCM extract without derivatization but at 520 nm for other extracts after derivatization with anisaldehyde sulphuric acid. The wavelengths for fingerprinting were selected by multiwavelength scanning showing the highest number of peaks.

The quantification of cucurbitacins B, D, and E; betulinic acid; picrosides 1 and 2; and apocynin was carried out in three different sets of chromatography for quality control of BAF.

Stock solutions of cucurbitacins B, D, and E; betulinic acid; picrosides 1 and 2; and apocynin were prepared in HPLC grade methanol to get a known concentration of 500 *μ*g mL^−1^. In the first set, the stock solutions of cucurbitacins B, D, and E were mixed in equal volume to get cucurbitacin standard (STC, 166.66 *μ*g mL^−1^ each). It was applied in triplicate in different volumes (0.1–10 *μ*L) on HPTLC plate and eluted using toluene : ethyl acetate : formic acid, 60 : 40 : 0.5, v/v/vas solvent system. The second set of chromatography was done for quantification of picrosides 1 and 2 and apocynin; similarly equal volumes of all three stock standards were mixed to get the picroside and apocynin standards (STPA, 166.66 *μ*g mL^−1^ each). This was also applied in triplicate in different volumes (0.1–10 *μ*L) on HPTLC plate and eluted and toluene : ethyl acetate : methanol : formic acid, 40 : 50 : 10 : 0.2, v/v/v/v, was used as solvent system. In the third set of chromatography, betulinic acid (STB) was applied as such (500 *μ*g mL^−1^) in triplicate in different volumes (0.1–10 *μ*L) and eluted, using same solvent as used for cucurbitacin. The sample (BAF, 8.0 *μ*L each) was applied in triplicate on every plate with the same chromatographic conditions as mentioned above. The quantification was done by scanning the developed chromatograms at 240 nm for cucurbitacins (without derivatization), at 595 nm for betulinic acid, and at 500 nm for picrosides and apocynin after derivatization with anisaldehyde sulphuric acid reagent.

### 2.8. Validation of the Method Developed

The newly developed HPTLC method was optimized and validated as per the ICH guidelines for calibration, linearity, precision, accuracy, robustness, specificity, LOD, and LOQ, similar to the other methods reported from this laboratory [35, 36].

### 2.9. Calibration Curve and Linearity

Different volumes (0.1–10 *μ*L spot^−1^) of the standard solutions (STC, STPA, and STB) were separately spotted on TLC plates (in triplicate) to obtain different concentrations of cucurbitacins, picrosides, apocynin (16.7–1666.6 ng spot^−1^), and betulinic acid (250–2500 ng spot^−1^) for calibration plots. The data of peak area versus drug concentration were treated by linear least square regression and the concentration range showing best regressions was considered for linearity [35].

### 2.10. Precision

Precision of the proposed method was obtained by repeatability and intermediate precision. Interday and intraday precisions were done by preparing and applying three different concentrations of standards (in triplicate) on the same day and on three different days, respectively. The interanalyst precision was carried out by repeating the same procedure using different systems of the same make and by different analysts, respectively. Precision studies were done at three different concentration levels. The method precision and intermediate precision were determined and reported in terms of % RSD [36].

### 2.11. Robustness of the Method

Robustness of the analytical procedure is a measure of its capacity to remain ineffective by small, but deliberate, variations in the method parameters and provide an indication of its reliability during normal usage. Robustness of the method was achieved by introducing small changes in the compositions of mobile phase and detection wavelength. The effect on the results was examined as % RSD [35].

### 2.12. Specificity

The specificity of the method was ascertained by analysing standard drug and sample. The detection of spots for cucurbitacins B, D, and E; betulinic acid; picrosides 1 and 2; and apocynin in BAF was confirmed by comparing *R*_*f*_ and spectra of spots with those of the standards. The peak purity was assessed by comparing the spectra at three different levels, that is, peak start, peak apex, and peak end positions of the spot [36].

### 2.13. Limit of Detection (LOD) and Limit of Quantification (LOQ)

The LOD was expressed as LOD = 3.3*σ*/slope, whereas LOQ was expressed as LOQ = 10*σ*/slope of calibration curve [36].

### 2.14. Accuracy as Recovery

In analytical methods the closeness of test results obtained by that method to the theoretical value is called the accuracy. The standard addition method was used by spiking at four different concentration levels, that is, 0, 50, 100, and 150%, of analyte in preanalyzed samples [36].

### 2.15. Analysis of Cucurbitacins B, D, and E; Betulinic Acid; Picrosides 1 and 2; and Apocynin in BAF

The newly developed method was applied for simultaneous estimation of cucurbitacins B, D, and E and betulinic acid as well as picrosides 1 and 2 and apocynin in DCM fraction of* P. kurroa*. The samples were applied in triplicate on HPTLC plates with standard and the contents of metabolites were analyzed, using regression equations obtained from calibration plots, and expressed as % w/w.

### 2.16. *In Vivo* Antitumor Activity on Balb/c Mice

The* in vivo* study was performed to carry out the anticancer activity of DCM fraction of hydroalcoholic (mother) extract of* P. kurroa* after oral administration to female Balb/c mice (25–30 g) as per the standard protocol [37–39]. The dose of DCM fraction was decided as per its extractive value equivalent to the dose of the drug (4.0 g per day) [37]. Animals were obtained from Central Animal Facility of Jamia Hamdard. This study was approved by and carried out under strict guidelines of Institutional Animal Ethics Committee (IAEC) of Jamia Hamdard, constituted by Committee for the Purpose of Control and Supervision of Experiments on Animals (CPCSEA, registration number 173/CPCSEA, 28 January 2000) of Ministry of Environment and Forest, Government of India (protocol approval number 915/22.10.2012).

### 2.17. Animals and Treatment Schedule

Twenty-four female Balb/c mice were procured from the central animal house facility of the University (Jamia Hamdard) and divided into four groups of six animals each. Group I, receiving 1% carboxymethyl cellulose (CMC) (0.2 mL oral, once daily for 10 days), served as control (nontumor mice, untreated); other groups received* Ehrlich ascites carcinoma* (EAC) cells (2 × 10^6^ cells/mouse, intraperitoneally (*i.p.*)), which was obtained generously from Cell Culture Laboratory of Dr. Dwarka Nath, INMAS, New Delhi. Group II served as toxic control (tumor induced, untreated mice), whereas group III received suspension of DCM fraction (50 mg Kg^−1^ body weight, orally) once daily for 10 d. However, group IV received standard 5-fluorouracil (20 mg Kg^−1^ body weight,* i.p.*) once daily for 10 d, after 24 h of EAC transplantation [38–40].

### 2.18. Analysis of Tumor Regression and Hematological Parameters after Oral Administration of DCM Fraction

The tumor regression parameters (tumor volume, packed cell volume, tumor weight, and viable and nonviable cell count) were analyzed after administration of last dose. The mice from each group were kept fasting for 18 h and blood samples were collected in ethylenediaminetetraacetic acid coated vials following anesthesia with ketamine-xylazine by cardiac puncture for the estimation of haematological toxicity. The animals were then sacrificed by cervical dislocation for the study of antitumor activity. Hematologic analysis was carried out using an automated hematologic analyzer (MS9 Differential Cell Counter 3 Part, HD Consortium, India). The mice were dissected and the peritoneal cavity was used to collect the ascetic fluid. The tumor volume was measured in a graduated centrifuge tube (in mL). The packed cell volume (PCV) was determined by centrifuging the ascetic fluid at 10,000 rpm for 5 minutes in centrifuge tube. This separates the fluid into layers. The volume of packed cells divided by the total volume of the ascetic fluid gives the % PCV. The tumor weight was calculated by measuring the weight (in gm) of mice before and after the collection of ascetic fluid from peritoneal cavity. The ascetic fluid was diluted 20 times with PBS, after which a drop of diluted cell suspension was placed on Neubauer's chamber and the number of cells was counted. The viability and nonviability of cells were checked by trypan blue assay. The viable and nonviable cells were counted as

(2) $$\begin{array}{c} \text{Cell count}=\frac{\left[\text{Number of cells}\times \text{Dilution factor}\right]}{\text{Area}\times \text{Thickness of the liquid film}}. \end{array}$$

The hematological parameters like total white blood cells (WBCs), red blood cell (RBC), lymphocytes (LYM), platelet (PLT), hematocrit (HCT), hemoglobin (HGB), mean corpuscular haemoglobin concentration (MCHC), mean corpuscular volume (MCV), mean corpuscular hemoglobin (MCH), red blood cell distribution width (RDW), and MID cells (less frequently occurring and rare cells correlating to basophils, monocytes, eosinophils, etc.) were determined using a blood automatic analyzer.

### 2.19. Statistical Analysis

Values were expressed as mean ± standard deviation (SD). One-way analysis of variance (ANOVA) followed by Dunnett's test (Graph Pad, San Diego, CA) was used for statistical analysis. All the treatment groups were compared with the toxic control group. *P* values < 0.05 were considered as statistically significant.

## 3. Results and Discussion

**Figure 1:**
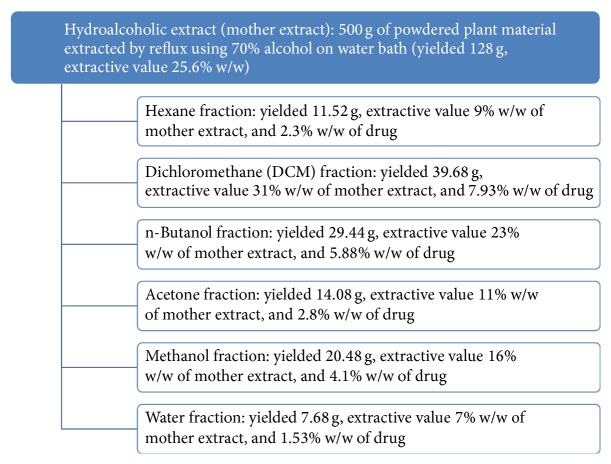
Schematic representation of extraction and fractionation of hydroalcoholic extract (mother extract) of* Picrorhiza kurroa* showing extractive values.

The plant material was extracted using crude alcohol (70%) by maceration and Reflux extraction after optimization. The hot extraction was selected for study due to its high yields and called mother extract (25.6% w/w). This was further fractionated using hexane (9% w/w), DCM (31% w/w), n-butanol (23% w/w), acetone (11% w/w), methanol (16% w/w), and water (7% w/w). However, 5.2 g of mother extract (4% w/w) of the drug was lost during the processing (Figure 1).

### 3.1. Cytotoxicity Assay

**Figure 2:**
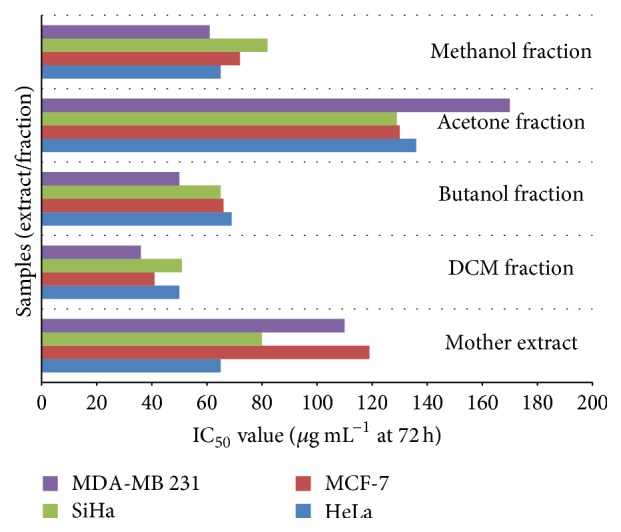
Bar graph showing IC_50_ of mother extract, DCM fraction, n-butanol fraction, acetone fraction, and methanol fraction at 72 h in HeLa, SiHa, MCF-7, and MDA-MB 231 cell lines.

**Figure 3:**
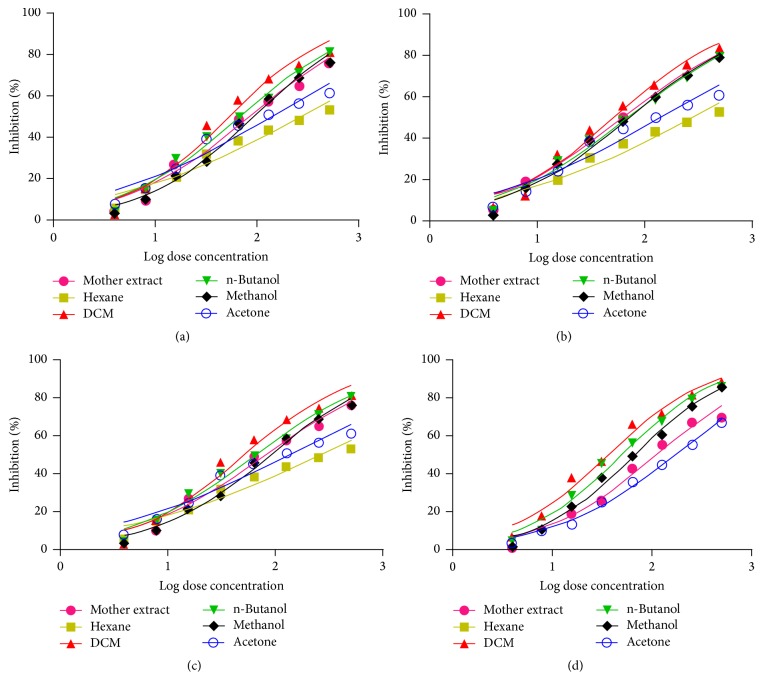
Dose response curve showing log dose concentration versus percent inhibition of extract/fractions of* P. kurroa* on cell lines at 72 h. (a) HeLa, (b) SiHa, (c) MCF-7, and (d) MDA-MB 231.

**Figure 4:**
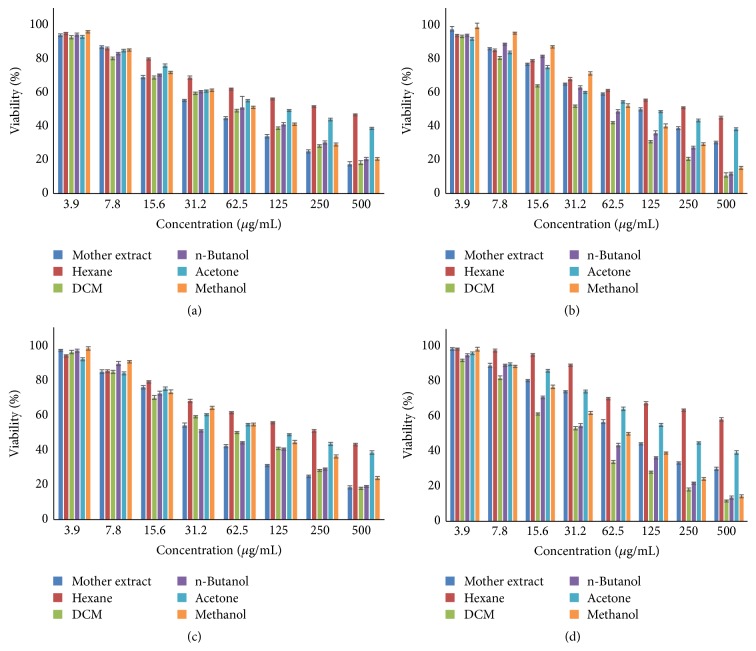
Bar graph showing concentration of extract/fractions of* P. kurroa* versus percent cell viability at 72 h. (a) HeLa, (b) MCF-7, (c) SiHa, and (d) MDA-MB 231 cell lines.

The cytotoxicity of hydroalcoholic (mother) extract and its fractions of* P. kurroa *on selected cancer cell lines was determined by MTT assay at 24, 48, and 72 h, which showed the best activity at 72 h. The results of cytotoxicity assay showed that hexane and water fractions did not produce any substantial cytotoxicity and were found safe in the tested concentration (500 *μ*g mL^−1^) in all cell lines. However, mother extract, DCM, n-butanol, acetone, and methanol fractions produced good cytotoxicity varying between 36 and 270 *μ*g mL^−1^ at 72 h among different cell lines (Figure 2). The DCM fraction (IC_50_ ranging from 36 to 51 *μ*g mL^−1^ at 72 h) showed best cytotoxic activities towards all cancer cell lines (Figures 3 and 4(a)–4(d)). The best cytotoxic activity of DCM fraction from* P. kurroa* may be attributed to the presence or synergistic activities of phytochemical components including sterol, triterpenes, and polyphenols [18, 20, 22–34]. However, betulinic acid may be attributed to this activity [41], since DCM fraction is rich in it, as proved by our analytical studies. MTT assay results of all the four cell lines proved that cytotoxicity was highest in DCM, followed by n-butanol, methanol fraction, and mother extract at 72 h (Figures 4(a)–4(d)). As the DCM fraction of hydroalcoholic extract of* P. kurroa* exhibited the highest cytotoxicity towards the tested cell lines, and this may be due to vacuole formation, membrane blebbing, nuclear condensation, and detachment of cells from the substratum and shrinkage of cells as well as development of apoptotic bodies [42, 43].

### 3.2. HPTLC Analysis

**Figure 5:**
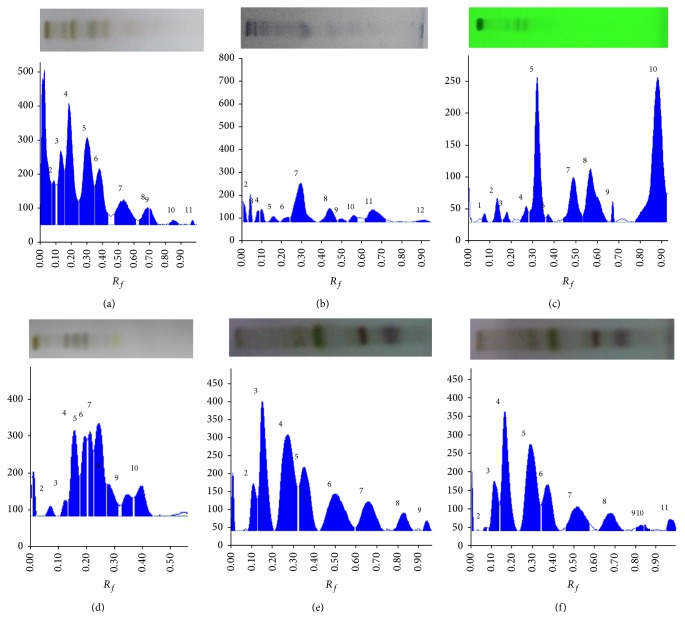
Developed TLC plates and HPTLC chromatogram. hydroalcoholic extract (a), hexane fraction (b), DCM fraction at 254 nm underivatized (c), n-butanol fraction (d), acetone fraction (e), and methanol fraction (f) of* P. kurroa* at 520 nm after derivatization with anisaldehyde sulphuric acid showing peaks of separated compounds.

The HPTLC fingerprinting of mother extract and different fractions was developed on silica gel. DCM fraction showed the maximum number of UV active compounds and was thus detected at 254 nm, whereas other fractions and mother extract were detected at 520 nm after visualization, using anisaldehyde sulphuric acid reagents (Figures 5(a)–5(f); Table 1). Table 1 showed different solvent systems used for fingerprinting of extract/fractions with number of spots present in them and their respective *R*_*f*_ values. The maximum number of compounds was observed in hexane and butanol fraction; however DCM fraction showed the presence of 8 UV active compounds.

### 3.3. Simultaneous Estimation of Cucurbitacins B, D, and E, Betulinic Acid, Picrosides 1 and 2, and Apocynin, Using Validated HPTLC Methods

**Figure 6:**
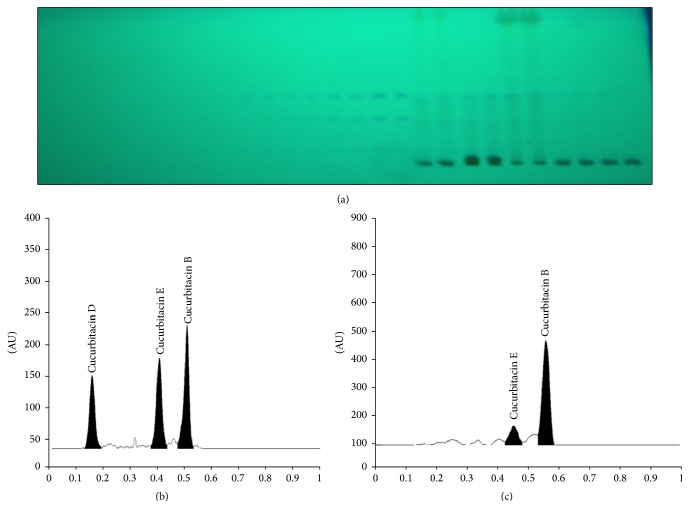
Developed HPTLC plate at 240 nm showing spots of cucurbitacins B, D, and E at different concentrations. Standard and in sample (a), HPTLC chromatograms of standard cucurbitacins B, D, and E (b), and sample (c).

**Figure 7:**
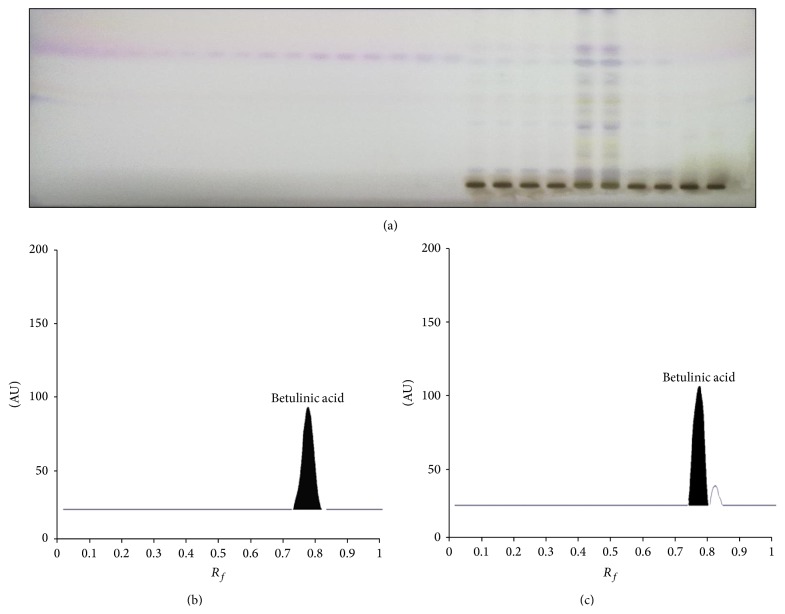
Developed HPTLC plate at 595 nm showing spots of betulinic acid at different concentrations. Standard and in sample (a), HPTLC chromatograms of standard betulinic acid (b), and sample (c).

**Figure 8:**
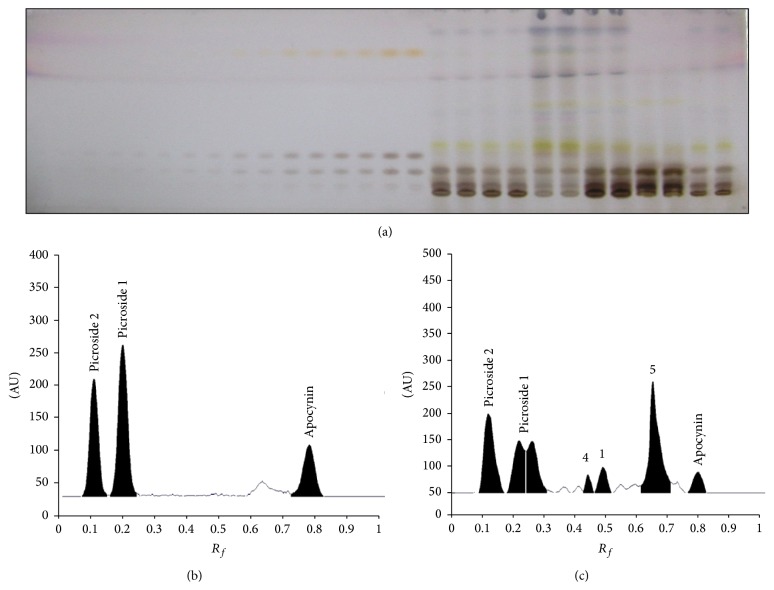
Developed HPTLC plate at 500 nm showing spots of picrosides 1 and 2 and apocynin at different concentrations. Standard and in sample (a), HPTLC chromatograms of standard picrosides 1 and 2 and apocynin (b), and sample (c).

The mobile phase toluene : ethyl acetate : formic acid (60 : 40 : 0.5, v/v/v) was optimized for simultaneous estimation of cucurbitacins B, D, and E, which showed good separation of all three compounds with compact peaks at different *R*_*f*_ values (0.53 ± 0.01, 0.16 ± 0.02, and 0.43 ± 0.01, resp.) (Figures 6(a)–6(c)) on scanning at 240 nm without derivatization. Betulinic acid was well separated, using the same solvent as indicated above for cucurbitacins, but visualized after derivatization using anisaldehyde sulphuric acid. It was scanned at 595 nm wavelength, which showed compact spot and sharp peak at *R*_*f*_  0.76 ± 0.01 (Figures 7(a)–7(c)). The toluene : ethyl acetate : methanol : formic acid (40 : 50 : 10 : 0.2, v/v/v/v) was optimized for separation and quantification of picrosides 1 and 2 and apocynin, which gave a good separation among components. The plate was scanned at 500 nm wavelengths after derivatization with anisaldehyde sulphuric acid, which produce very well defined peaks of picrosides 1 and 2 and apocynin at *R*_*f*_ values 0.23 ± 0.01, 0.11 ± 0.02, and 0.77 ± 0.01, respectively (Figures 8(a)–8(c)).

### 3.4. Validation of the Method Developed

#### 3.4.1. Calibration Curve and Linearity

The newly developed methods for simultaneous estimation of cucurbitacins B, D, and E; betulinic acid; picrosides 1 and 2; and apocynin were found linear for a wide range of concentration with good regression coefficient (>0.99). The linearity data of all the biomarkers developed such as range linearity, regression equation, regression coefficient, slope, intercept, LOD, and LOQ are given in Table 2.

#### 3.4.2. Precision

The method precision and intermediate precisions were determined and reported in terms of % RSD. Precision of the proposed method was obtained by repeatability and intermediate precision at three different concentration levels. The % RSD of interday precision, intraday precision, and interanalyst precision was within the range of 1.61–2.12 for all compounds, as reported in Table 3.

#### 3.4.3. Robustness of the Method

The low values of % RSD obtained after introducing small but deliberate changes in mobile phase composition and wavelength indicated robustness of the methods (Tables 4(a)–4(c)) at 3 different concentration levels.

#### 3.4.4. Specificity

The specificity of the methods was ascertained by analysing standard drugs and samples. The detection of spot for cucurbitacins B, D, and E; betulinic acid; picrosides 1 and 2; and apocynin in DCM sample was confirmed by equating *R*_*f*_ and spectra of spot with the standard. The peak purity was estimated by comparing the spectra at three different levels, that is, at peak start, peak apex, and peak end positions of the spot.

#### 3.4.5. Limit of Detection (LOD) and Limit of Quantification (LOQ)

The LOD and LOQ of different markers were calculated as per the standard protocol [36, 37] and reported in Table 3. The LOD of markers lies in the range of 21.94–133.0 ng, indicating good sensitivity of methods for simultaneous quantification of compounds.

#### 3.4.6. Accuracy as Recovery

The accuracy was calculated as recovery by standard addition method by spiking 0, 50, 100, and 150% of analyte in preanalyzed samples, showing good recovery of all biomarkers used and lying in the range of 99–101.4% (Table 5).

#### 3.4.7. Estimation of Cucurbitacins B, D, and E; Betulinic Acid; Picrosides 1 and 2; and Apocynin in DCM Fraction

The newly developed and validated HPTLC method was applied for the analysis of cucurbitacins B, D, and E; betulinic acid; picrosides 1 and 2; and apocynin in DCM fraction of* P. kurroa* rhizome. The peak areas of triplicate samples were analyzed by regression equation obtained from the calibration plot. The content obtained for different markers is reported in Table 6. Cucurbitacin D was found absent in DCM fraction (BAF) of* P. kurroa*.

### 3.5. *In Vivo *Anticancer Activity of DCM Fraction

**Figure 9:**
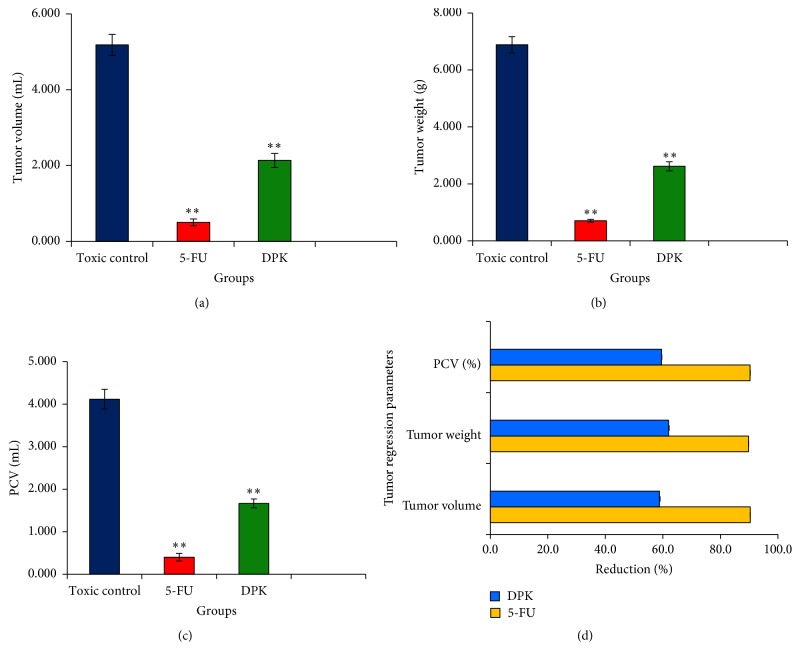
Showing tumor regression parameters as obtained on Balb/c mice after ten days of oral administration of DCM fraction of hydroalcoholic extract of* P. kurroa* (*∗∗* showed *p* < 0.01 followed by Dunnett's test in comparison to EAC control).

DCM fraction of* P. kurroa* showed a significant effect on tumor regression parameters of EAC cell bearing mice. The DCM fraction significantly (*P* < 0.01) reduced the tumor volume, tumor weight, and % packed cell volume at a dose of 50 mg Kg^−1^ body weight, as compared with EAC (toxic) control group (Figure 9). The results were almost comparable to that of 5-FU, a standard marketed drug. There was a significant decrease in number of tumor cells on treatment with DCM fraction and 5-FU in tumor-bearing mice, as compared with EAC control. Similarly, a percentage of viable cells were decreased significantly in treatment groups, as compared with untreated EAC control (Table 7).

Haematological parameters of EAC tumor-bearing and treatment group mice were studied on day 14, which showed significant changes in the number of WBCs only, and that was reversed in treated groups as compared with untreated EAC control. Other parameters such as haemoglobin, RBC, lymphocytes, hematocrit (HCT), RDW, and PLT were found to be near normal and did not produce any significant alteration (Table 8).

The well-founded criteria for assessing the value of any anticancer drug are the increase in life span, the loss of leukemic cells from the blood, and reduction of solid tumor volume. Transplantable tumor cells, such as EAC, are rapidly growing cancer cells with aggressive behavior [37–39]. The tumor implantation includes a local inflammatory reaction by increasing vascular permeability and results in an intense ascetic fluid accumulation [37, 39]. Our results showed significant reversal of tumor regression parameters accompanied by a reduction in WBC count after treatment with DCM fraction of hydroalcoholic extract of* P. kurroa*. The best active/enriched fraction also inhibited the accumulation of ascetic fluid in the peritoneal cavity of the tumor-bearing animals. These results clearly demonstrated the antitumor effect of* P*.* kurroa* on EAC tumor cells.

## 4. Conclusion

This study has indicated that hydroalcoholic (mother) extract and its medium polar fractions of* P. kurroa* exhibited cytotoxic potential, while water and hexane fractions did not produce cytotoxicity in cervical and breast cancer cell lines up to 500 *μ*g mL^−1^ and 72 h. The DCM fraction was found as best active fraction in* in vitro* testing with lowest IC_50_ value (36–51 *μ*g mL^−1^ at 72 h) among the four tested cell lines. This might be due to the presence of cucurbitacins B and E; betulinic acid; picrosides 1 and 2; and apocynin, as obtained from analytical studies and supported by earlier reports [15, 18–21]. The analysis of seven markers (six terpenoid and one flavonoid) for quality control of DCM fraction using simultaneous HPTLC methods in present investigation is unique and being reported for the first time. The oral administration of DCM fraction (BAF) of hydroalcoholic (mother) extract of* P. kurroa* (50 mg Kg^−1^) in Balb/c mice reduced the tumor volume and weight and % packed cell volume as well as WBC reflecting antitumor activity of* P. kurroa*. Our results suggest that DCM fraction of hydroalcoholic extract of* P. kurroa* might be a good candidate for development as anticancer drug and may come out as a new future phytopharmaceutical drug since inclusion of phytopharmaceuticals/enriched fractions is already in the process in several pharmacopoeias. In addition, simultaneous methods developed and validated for quantification of cucurbitacin (B, D, and E), betulinic acid, picroside 1, picroside 2, and apocynin can be used for its quality control as well as for that of other drugs containing them as ingredient.

## Figures and Tables

**Table 1:** HPTLC fingerprint data of mother extract and different fractionation of *P. kurroa*.

Extracts	Solvent system	Visualization	Number of major spots and *R*_*f*_ values
Hydroalcoholic extract/mother extract	Methanol : ethyl acetate : formic acid (0.5 : 5 : 1, v/v/v)	Anisaldehyde sulphuric acid at 520 nm	(07) 0.11, 0.16, 0.25, 0.35, 0.47, 0.63, 0.96.
Hexane fraction	Toluene : ethyl acetate (1.8 : 0.1, v/v)	Anisaldehyde sulphuric acid at 520 nm	(09) 0.09, 0.13, 0.19, 0.24, 0.38, 0.48, 0.53, 0.61, 0.87
DCM fraction	Toluene : ethyl acetate : formic acid (1.5 : 0.6 : 0.1, v/v/v)	At 254 nm	(08) 0.11, 0.16, 0.24, 0.28, 0.44, 0.53, 0.66, 0.77
n-Butanol fraction	Methanol : ethyl acetate : formic acid (0.5 : 5 : 1, v/v/v)	Anisaldehyde sulphuric acid at 520 nm	(09) 0.10, 0.13, 0.18, 0.20, 0.23, 0.27, 0.32, 0.37.
Acetone fraction	Methanol : ethyl acetate : formic acid (0.5 : 5 : 1, v/v/v)	Anisaldehyde sulphuric acid at 520 nm	(07) 0.08, 0.14, 0.24, 0.34, 0.45, 0.62, 0.80.
Methanol fraction	Methanol : ethyl acetate : formic acid (0.5 : 5 : 1, v/v/v)	Anisaldehyde sulphuric acid at 520 nm	(08) 0.08, 0.13, 0.22, 0.33, 0.43, 0.60, 0.67, 0.79

**Table 2:** Linearity data of chromatographic HPTLC method for cucurbitacins B, D, and E; betulinic acid; picrosides 1 and 2; and apocynin (*n* = 3).

S. number	Biomarkers	Solvent system	Linearity (ng spot^−1^)	Equation	Regression ± SD	Slope ± SD	Intercept ± SD	LOD (ng spot^−1^)	LOQ (ng spot^−1^)
1	Cucurbitacin B	(Toluene : ethyl acetate : formic acid 60 : 40 : 0.5, v/v/v)	66.7–833.3	*Y* = 340.18 + 2.7*x*	0.995 ± 0.001	2.70 ± 0.006	340.18 ± 0.040	22.06	66.86
2	Cucurbitacin D		166.6–833.33	*Y* = 80.76 + 1.89*x*	0.996 ± 0.001	1.89 ± 0.001	80.75 ± 0.020	54.85	166.20
3	Cucurbitacin E		166.6–833.33	*Y* = 200.09 + 2.33*x*	0.998 ± 0.001	2.33 ± 0.006	200.76 ± 0.121	55.00	166.69
4	Betulinic acid		250–2500	*Y* = 827.64 + 0.504*x*	0.993 ± 0.002	0.504 ± 0.004	827.64 ± 0.10	82.5	250.0

5	Picroside 1	(Toluene : ethyl acetate : methanol : formic acid 40 : 50 : 10 : 0.5, v/v/v/v)	66.7–833.33	*Y* = 565.75 + 5.00*x*	0.994 ± 0.001	5.00 ± 0.002	565.8 ± 0.0133	21.97	66.58
6	Picroside 2		67.7–833.33	*Y* = 234.86 + 3.66*x*	0.996 ± 0.002	3.66 ± 0.045	234.86 ± 0.303	22.28	67.53
7	Apocynin		333.33–1666.66	*Y* = 152.97 + 1.879*x*	0.991 ± 0.001	1.87 ± 0.031	152.97 ± 1.25	110.18	333.88

**Table 3:** Precision of the method for the estimation of cucurbitacins B, D, and E; betulinic acid; picrosides 1 and 2; and apocynin (*n* = 6).

Conc. (ng^a^ spot^−1^)	Interday precision		Intraday precision		Interanalyst precision	
	Mean peak area ± SD^b^	% RSD^c^	Mean peak area ± SD	% RSD	Mean peak area ± SD	% RSD
Cucurbitacin B						

100	541.00 ± 10.53	1.94	552.0 ± 10.81	1.95	541.0 ± 11.00	2.03
200	860.66 ± 15.04	1.74	875.0 ± 16.50	1.88	859.66 ± 16.62	1.93
400	1585.0 ± 26.72	1.68	1594.66 ± 25.71	1.61	1580.66 ± 29.36	1.85

Cucurbitacin D						

200	461.33 ± 10.01	2.17	470.00 ± 10.00	2.12	442.66 ± 8.73	1.97
400	825.00 ± 15.52	1.88	837.33 ± 15.94	1.90	838.33 ± 16.04	1.91
800	1634.33 ± 32.12	1.96	1647.33 ± 33.29	2.02	1652.06 ± 34.59	2.09

Cucurbitacin E						

200	637.33 ± 13.01	2.04	641.0 ± 12.66	1.97	632.66 ± 12.85	2.03
400	1157.33 ± 19.75	1.70	1172.66 ± 20.40	1.73	1162.66 ± 21.54	1.85
800	2138.0 ± 42.22	1.97	2135.66 ± 46.36	2.17	2135.33 ± 40.07	1.87

Betulinic acid						

250	979.66 ± 18.50	1.88	976 ± 20.59	2.10	981.33 ± 19.55	1.99
500	1039.66 ± 21.00	2.02	1022.66 ± 19.55	1.91	1031.33 ± 20.03	1.94
1000	1374 ± 25.89	1.88	1375.66 ± 25.53	1.85	1387.33 ± 26.63	1.91

Picroside 1						

100	941.00 ± 17.50	1.84	944.0 ± 19.50	2.05	943.0 ± 17.08	1.81
200	1642.66 ± 33.5	2.04	1639.33 ± 30.92	1.88	1644.33 ± 32.86	1.99
400	2936.0 ± 56.95	1.93	2940.0 ± 56.66	1.92	2929.33 ± 59.65	2.03

Picroside 2						

100	502.0 ± 9.53	1.90	504.66 ± 10.50	2.08	506.0 ± 9.16	1.81
200	1005.66 ± 18.0	1.84	1008.33 ± 18.94	1.82	1007.33 ± 20.10	1.99
400	1916.66 ± 33.08	1.72	1919.33 ± 37.01	1.92	1922.66 ± 40.69	2.11

Apocynin						

400	795.0 ± 15.01	1.88	799.0 ± 15.52	1.94	803.00 ± 15.09	1.88
800	1583.33 ± 31.0	1.95	1589.33 ± 32.25	2.02	1594.33 ± 31.65	1.98
1000	2225.66 ± 45.93	2.05	2231.33 ± 41.63	1.86	2227.33 ± 40.50	1.81

^a^Nanogram. ^b^Standard deviation. ^c^Relative standard deviation.

**(a):** 

Parameters				Mean area ± SD^b^	% RSD^c^ of area
	Components	Conc. (ng spot^−1^)	Wavelength used		
Detecting wavelengths (nm^a^) 240 and 595	Cucurbitacin B	100	238	554.33 ± 11.67	2.10
			242	545.66 ± 10.78	1.97
		200	238	875.66 ± 19.55	2.23
			242	876.66 ± 18.08	2.06
		400	238	1594.66 ± 25.71	1.61
			242	1588.66 ± 32.00	2.01
	Cucurbitacin D	200	238	441.0 ± 9.00	2.04
			242	438.33 ± 9.07	2.07
		400	238	830.33 ± 16.55	1.98
			242	824.33 ± 16.25	1.97
		800	238	1648.00 ± 32.42	1.97
			242	1650.33 ± 32.00	1.94
	Cucurbitacin E	200	238	640.66 ± 13.86	2.16
			242	638.00 ± 13.00	2.03
		400	238	1173.33 ± 22.03	1.94
			242	1169.33 ± 22.05	1.88
		800	238	2161.33 ± 41.24	1.91
			242	2154.33 ± 43.85	2.01
	Betulinic acid	250	593	981.33 ± 19.55	1.99
			597	977.33 ± 18.87	1.93
		500	593	1039.66 ± 21.00	2.02
			597	1031.00 ± 18.35	1.78
		1000	593	1363.66 ± 27.09	1.98
			597	1368.33 ± 27.61	2.01

Detecting wavelength (nm^a^) 500	Picroside 1	100	498	941.33 ± 17.50	1.85
			502	946.00 ± 17.34	1.83
		200	498	1645.00 ± 34.05	2.06
			502	1644.33 ± 32.86	1.99
		400	498	2927.00 ± 55.05	1.88
			502	2940.00 ± 56.66	1.92
	Picroside 2	100	498	504.00 ± 10.00	1.98
			502	502.00 ± 9.53	1.90
		200	498	1004.00 ± 20.42	2.03
			502	1007.33 ± 19.50	1.93
		400	498	1919.33 ± 37.01	1.92
			502	1922.66 ± 40.69	2.11
	Apocynin	400	498	799.00 ± 15.50	1.94
			502	797.00 ± 14.54	1.82
		800	498	1593.33 ± 29.36	1.84
			502	1594.33 ± 31.65	1.98
		1000	498	2227.33 ± 40.50	1.81
			502	2257.00 ± 43.13	1.92

^a^Nanometer. ^b^Standard deviation. ^c^Relative standard deviation.

**(b):** 

Parameters			Mean area ± SD^a^	% RSD^b^ of area
Components	Conc. (ng spot^−1^)	Mobile phase composition (toluene : ethyl acetate : formic acid, 60 : 40 : 0.5, v/v/v)		
Cucurbitacin B	100	63 : 37 : 0.5	545.0 ± 11.78	2.16
		61 : 39 : 0.5	552.0 ± 12.00	2.17
		57 : 43 : 0.5	544.0 ± 10.44	1.91
	200	63 : 37 : 0.5	872.33 ± 16.28	1.86
		61 : 39 : 0.5	868.33 ± 16.25	1.87
		57 : 43 : 0.5	873.33 ± 16.56	1.91
	400	63 : 37 : 0.5	1605.0 ± 28.16	1.75
		61 : 39 : 0.5	1590.33 ± 26.33	1.67
		57 : 43 : 0.5	1603.66 ± 30.66	1.91

Cucurbitacin D	200	63 : 37 : 0.5	437.0 ± 9.00	2.06
		61 : 39 : 0.5	440.67 ± 9.5	2.16
		57 : 43 : 0.5	442.33 ± 10.07	2.28
	400	63 : 37 : 0.5	826.33 ± 15.04	1.82
		61 : 39 : 0.5	828.33 ± 17.01	2.05
		57 : 43 : 0.5	831.33 ± 15.72	1.89
	800	63 : 37 : 0.5	1644.00 ± 36.05	2.28
		61 : 39 : 0.5	1647.00 ± 29.82	1.81
		57 : 43 : 0.5	1643.66 ± 38.22	2.33

Cucurbitacin E	200	63 : 37 : 0.5	641.33 ± 12.34	1.92
		61 : 39 : 0.5	645.33 ± 12.01	1.86
		57 : 43 : 0.5	638.00 ± 12.53	1.96
	400	63 : 37 : 0.5	1176.67 ± 23.71	2.02
		61 : 39 : 0.5	1172.33 ± 22.59	1.93
		57 : 43 : 0.5	1170.33 ± 23.24	1.98
	800	63 : 37 : 0.5	2149.67 ± 40.55	1.89
		61 : 39 : 0.5	2154.0 ± 43.51	2.02
		57 : 43 : 0.5	2156.33 ± 44.0	2.04

Betulinic acid	250	63 : 37 : 0.5	978.0 ± 17.78	1.81
		61 : 39 : 0.5	977.33 ± 18.87	1.93
		57 : 43 : 0.5	972.33 ± 19.85	2.04
	500	63 : 37 : 0.5	1033.00 ± 21.79	2.10
		61 : 39 : 0.5	1022.66 ± 17.50	1.71
		57 : 43 : 0.5	1037.33 ± 18.92	1.82
	1000	63 : 37 : 0.5	1364.66 ± 25.69	1.88
		61 : 39 : 0.5	1371.00 ± 26.05	1.90
		57 : 43 : 0.5	1363.66 ± 27.09	1.98

^a^Standard deviation. ^b^Relative standard deviation.

**(c):** 

Parameters			Mean area ± SD^a^	% RSD^b^ of area
Components	Conc. (ng spot^−1^)	Mobile phase composition (toluene : ethyl acetate : methanol : formic acid, 40 : 50 : 10 : 0.5, v/v/v/v)		
Picroside 1	100	42 : 48 : 10 : 0.2	946.00 ± 17.34	1.83
		38 : 52 : 10 : 0.2	948.33 ± 17.50	1.84
		40 : 52 : 08 : 0.2	948.66 ± 18.00	1.89
	200	42 : 48 : 10 : 0.2	1645.00 ± 34.04	2.06
		38 : 52 : 10 : 0.2	1643.00 ± 31.43	1.91
		40 : 52 : 08 : 0.2	1643.66 ± 30.66	1.86
	400	42 : 48 : 10 : 0.2	2927.00 ± 55.05	1.88
		38 : 52 : 10 : 0.2	2931.66 ± 60.87	2.07
		40 : 52 : 08 : 0.2	2927.66 ± 60.17	2.05

Picroside 2	100	42 : 48 : 10 : 0.2	504.00 ± 10.00	1.98
		38 : 52 : 10 : 0.2	501.33 ± 9.81	1.95
		40 : 52 : 08 : 0.2	506.66 ± 9.71	1.91
	200	42 : 48 : 10 : 0.2	1007.33 ± 19.50	1.93
		38 : 52 : 10 : 0.2	1005.66 ± 19.85	1.97
		40 : 52 : 08 : 0.2	1004.00 ± 20.42	2.03
	400	42 : 48 : 10 : 0.2	1914.33 ± 33.50	1.75
		38 : 52 : 10 : 0.2	1922.66 ± 40.68	2.11
		40 : 52 : 08 : 0.2	1915.33 ± 37.68	1.96

Apocynin	400	42 : 48 : 10 : 0.2	800.66 ± 15.94	1.99
		38 : 52 : 10 : 0.2	801.66 ± 15.63	1.94
		40 : 52 : 08 : 0.2	797.00 ± 14.52	1.82
	800	42 : 48 : 10 : 0.2	1593.33 ± 29.36	1.84
		38 : 52 : 10 : 0.2	1587.66 ± 28.67	1.80
		40 : 52 : 08 : 0.2	1597.00 ± 30.61	1.91
	1000	42 : 48 : 10 : 0.2	2222.66 ± 42.44	1.90
		38 : 52 : 10 : 0.2	2257.00 ± 43.13	1.91
		40 : 52 : 08 : 0.2	2228.00 ± 44.22	1.98

^a^Standard deviation. ^b^Relative standard deviation.

**Table 5:** Accuracy of the HPTLC methods for the estimation of cucurbitacins B and E, betulinic acid, picrosides 1 and 2, and apocynin.

% of standard spiked to the sample	Theoretical content (*µ*g mL^−1^)	Amount of drug recovered (*µ*g mL^−1^)	% of drug recovered	% RSD
Cucurbitacin B				
0	300	299.66	99.88	0.51
50	450	450.66	100.14	0.34
100	600	600.67	100.11	0.25
150	750	751.33	100.17	0.07
Cucurbitacin E				
0	70	71.0	101.42	1.40
50	105	105.0	100.00	0.95
100	140	141.0	100.71	0.70
150	175	175.33	100.19	0.32
Cucurbitacin D				
0	250	248.58	99.43	0.17
50	350	351.15	100.3	0.58
100	500	498.21	99.64	0.36
150	650	647.24	99.57	0.5
Betulinic acid				
0	34	33.83	99.50	1.03
50	51	51.5	100.98	0.97
100	68	68.96	101.42	0.65
150	85	85.43	100.50	0.99
Picroside 1				
0	50	50.66	101.33	1.13
50	75	75.0	100.00	1.33
100	100	100.0	100.00	1.00
150	125	125.0	100.26	0.92
Picroside 2				
0	130	131.66	101.28	0.43
50	195	196.00	100.51	0.52
100	260	261.66	100.64	0.22
150	325	324.66	99.89	0.17
Apocynin				
0	50	49.33	98.66	1.17
50	75	74.33	99.11	0.77
100	100	99.0	99.00	1.01
150	125	124.66	99.73	0.46

**Table 6:** Estimation of cucurbitacins B and E, betulinic acid, picrosides 1 and 2, and apocynin in DCM fraction of *P. kurroa*.

Components	% yield from DCM fraction of *P. kurroa *%w/w
Cucurbitacin B	2.98 ± 0.051
Cucurbitacin E	0.707 ± 0.004
Cucurbitacin D	Nil
Betulinic acid	3.43 ± 0.351
Picroside 1	0.537 ± 0.030
Picroside 2	1.33 ± 0.036
Apocynin	0.526 ± 0.004

**Table 7:** Tumor cell count of groups (groups II, III, and IV) (mean ± SD, *n* = 3).

Groups	Total cells/mL *∗* 10^7^ ± SD	Viable cells/mL *∗* 10^7^ ± SD	Nonviable cells/mL *∗* 10^7^ ± SD	% viable cells ± SD	% nonviable cells ± SD
EAC	9.93 ± 0.81	9.43 ± 0.57	0.50 ± 0.59	95.22	4.77
5-FU	2.91 ± 0.33^*∗∗*^	0.53 ± 0.17^*∗∗*^	2.38 ± 0.31^*∗∗*^	18.28^*∗∗*^	81.71^*∗∗*^
DPK	4.96 ± 0.54^*∗∗*^	2.31 ± 0.43^*∗∗*^	2.65 ± 0.74^*∗∗*^	47.23^*∗∗*^	52.76^*∗∗*^

EAC: Ehrlich ascites carcinoma, 5-FU: 5-fluorouracil, DPK: dichloromethane fraction of *P. kurroa* (*∗∗* showed *P* < 0.01 followed by Dunnett's test in comparison to EAC control).

**Table 8:** Comparative haematological profile of EAC, control, standard, and DCM fraction treated groups of Balb/C mice after ten days of treatment.

Parameters	Control ± SD	EAC control ± SD	5-FU ± SD	DPK ± SD
WBC *∗* 10^3^/*µ*L	5.20 ± 0.26	9.50 ± 0.47	3.10 ± 0.15^*∗∗*^	0.55 ± 0.02^*∗∗*^
RBC *∗* 10^6^/*µ*L	9.36 ± 0.46	7.29 ± 0.36	8.79 ± 0.44	8.42 ± 0.42
HGB g/dL	13.40 ± 0.67	10.70 ± 0.53	13.00 ± 0.65	11.30 ± 0.56
HCT %	45.80 ± 2.29	36.30 ± 1.81	45.00 ± 2.25	42.50 ± 2.12
MCV fL	48.90 ± 2.44	49.80 ± 2.49	51.20 ± 2.56	51.20 ± 2.56
MCH pg	14.30 ± 0.71	14.70 ± 0.73	14.80 ± 0.74	14.30 ± 0.71
MCHC g/dL	29.30 ± 1.46	29.30 ± 1.46	28.90 ± 1.44	29.20 ± 1.46
PLT *∗* 10^5^/*µ*L	7.08 ± 0.35	11.84 ± 0.59	6.83 ± 0.34	8.16 ± 0.41
RDW fL	29.20 ± 1.46	29.90 ± 1.49	30.40 ± 1.52	29.30 ± 1.46
PDW fL	9.10 ± 0.45	10.40 ± 0.52	9.00 ± 0.45	9.10 ± 0.45
MPV fL	7.40 ± 0.37	7.90 ± 0.39	6.90 ± 0.34	7.50 ± 0.37
P-LCR %	9.80 ± 0.49	11.90 ± 0.59	6.10 ± 0.30	10.20 ± 0.51

*∗∗* showed *P* < 0.01 followed by Dunnett's test in comparison to EAC control.
